# Aus der Schockstarre zur Akteurszentrierung – die Phasen der Pandemie-Berichterstattung

**DOI:** 10.1007/s41358-021-00260-9

**Published:** 2021-05-04

**Authors:** Matthias Degen

**Affiliations:** Institut für Journalismus und Public Relations, Westfälische Hochschule, Neidenburger Str. 43, 45897 Gelsenkirchen, Deutschland

Es ist die Zeit der Premieren: Größtmögliche Distanz, größtmögliche Geschwindigkeit, größtmögliche Verunsicherung. Die Pandemie versetzt Politik und Journalismus gleichermaßen in einen Krisenmodus. In dieser Situation stellt sich die Handlungslage der politischen Akteure weithin unsortiert dar. Die Politik ist getrieben von immer neuen Eskalationsstufen der Pandemie, der wissenschaftliche Diskurs liefert eng getaktet Befunde, und Journalistinnen und Journalisten sind zunächst überfordert vom Tempo der Ereignisse, versuchen abzubilden, was in Hyper-Geschwindigkeit entschieden wurde.

Eine Bundeskanzlerin, sechzehn Ministerpräsidentinnen und Kollegen, dazu die Adjutanten aus allen politischen Lagern, die gern in alle erdenklichen Richtungen ziehen und zerren. Erwähnt sei beispielhaft ein Karl Lauterbach, der als Gesundheitsexperte ohne Amt quasi als Gegenpapst bisweilen zum RKI-Präsidenten und bisweilen zum Gesundheitsminister auftritt. Das Ziehen und Zerren an sich ist noch nichts Ungewöhnliches und könnte als Nukleus einer funktionierenden demokratischen Meinungsbildung oder auch erweiterten Gewaltenteilung verstanden werden. Wenn die Crux nicht darin läge, dass das Virus sich nicht an Zuständigkeiten hielte und alle irgendwie beträfe. So reden also alle ein wenig mit, zementieren ihre Fürstentümer und liefern den Stoff für eine tragische Soap-Opera, an deren unendliche Fortsetzung sich die meisten Handelnden schon gewöhnt haben. So auch die Beobachterinnen und Kommentatoren.

Der Journalismus nimmt in der Pandemie seine Aufgabe und Funktion als Vermittler, Kontrolleur und Diskurskatalysator öffentlicher Meinungsbildungsprozesse zwar an, scheitert aber anfangs am reflexionsfreien Raum der Echtzeit-Kommunikation. Es wird getickert, was das Zeug hält. Bald könnte man meinen, die Corona-Pandemie fällt ins Transfer-Fenster der Bundesliga und Politikjournalisten orientieren sich am Push-News-Gewitter des Boulevards. Sendezeit und Titelseiten, die für die Corona-Pandemie verwendet werden, verdrängen andere Sachverhalte und Ereignisse aus dem Sichtfeld.

## Phasen der Pandemie-Berichterstattung

Verschiedene Phasen der Pandemie bringen zugleich auch verschiedene Phasen politischer Kommunikation und Politikberichterstattung hervor: Spätestens mit den Bildern des LKW-Konvois der italienischen Armee, der Särge von COVID-19-Toten aus Bergamo abtransportiert, beginnt die *Phase der Schockstarre*. Corona dominiert die Berichterstattung, die Nachrichten werden monothematisch, es ist die Zeit der Sondersendungen in Dauerschleife (Eisenegger et al. 2020). Ein Reaktionsmuster, das aus der Katastrophenberichterstattung bekannt ist und den Redaktionen einen Adrenalinschub versetzt. Der Ton wird ernst, die Konzentration richtet sich auf die Regierenden, in Erwartung auf Antworten, die berichtet, beraten, bewertet werden können. Eine Phase, die sich auf das Verstehen und Erklären ausrichtet, auf das Sortieren einer Lage. Journalistische Kernaufgaben wie das Hinterfragen, In-Frage-Stellen und Einfordern von Handlungsbegründungen treten in den Hintergrund (Ruß-Mohl 2020). Neben den Regierenden treten Expertinnen und Experten in Erscheinung, allerdings ist der Ausschnitt der Wissenschaft weitgehend reduziert auf die Welt der Virologen, andere in der Pandemie relevante Disziplinen finden zunächst nicht den Weg in den medialen Diskurs (Wormer 2020).

Die für das Funktionieren eines öffentlichen Diskurses unabdingbaren Aufgaben und Themenweitungen finden zurück, als die Krise unter Kontrolle scheint, die Berichterstattung damit den Weg aus der Notaufnahme zur Weiterbehandlung antritt: die *Phase der Rückbesinnung auf Kritik*. Reine Informationsorientierung als Krisenreaktionsmuster des Journalismus wurde nach den ersten Wochen abgelöst von einer multiperspektivischen und facettenreichen Berichterstattung über die Krise (Quandt et al. 2020). Der Blick richtet sich wieder auf Kritiker, auf den Ausgleich der Grundrechte und auf die Verlierer der Krise. Mit sinkenden Infektionszahlen beginnt die Debatte um Lockerungen der Corona-Maßnahmen, es schlägt die Stunde der Opposition auch im medialen Diskurs. Alternative Politikangebote vor allem von FDP und Grünen weiten den Diskurs und werden den Groß-Koalitionären und Ministerpräsidentinnen in all ihrer Uneinigkeit (Laschet vs. Söder) vorgehalten. Das Einfordern von Begründungen für Maßnahmen und der Abgleich der Notwendigkeiten wird damit nicht allein der „Querdenken“-Bewegung überlassen, sondern findet zurück in den demokratischen und vernunftorientierten Diskurs der Massenmedien, die parallel zudem einen Schnellkurs „How-to-Wissenschaft“ für die geneigte Öffentlichkeit anbieten. Selten fand soviel service-orientierter Erklär-Journalismus in die Sendepläne und Content-Management-Systeme, der Hygiene, Ansteckungsmuster, Krankheitsverläufe, Impfungen und Statistiklehre profund vermittelte. Plötzlich waren Begriffe wie R‑Wert, Inzidenzen, Aerosole und Antikörper geläufige Small-Talk-Themen für die Mittagspause.

Spätestens mit dem Jahreswechsel 2020/2021 richtet sich die Aufmerksamkeit verstärkt auf die Parteienebene und deren individuelle Leistungsfähigkeit, zur Problemlösung beizutragen. Die digitale Live-TV-Show des CDU-Bundesvorsitzenden mag als Startschuss verstanden werden. Wer macht es, wenn Angela Merkel abtritt? Wer kann es? Bröckelt das Unions-Bollwerk? Welche Partei versteht es, rechtschaffen durch die Krise zu manövrieren und glaubwürdige Führungsangebote zu unterbreiten? Diese Fragen stehen fortan gleichberechtigt neben den Alltagsfragen um Lockdown oder Lockerung. *Die Phase der parteipolitischen Akteurszentrierung *prägt die Berichterstattung. In der Politikberichterstattung schafften es schließlich die Landtagswahlen in Rheinland-Pfalz und Baden-Württemberg, Corona kurzfristig auf Seite 2 zu verweisen. Zugleich haben sie zu Beginn des Superwahljahrs 2021 einen Vorgeschmack auf den Alltag der Distanzdemokratie im Rahmen der Pandemie geliefert. Kleintaktige Entscheidungen der politischen Akteure werden gemessen an politischen Grundsätzen. Gravierende individuelle Verfehlungen wie die Bereicherung einzelner Unions-Abgeordneter am Geschäft mit Masken treffen einem Bumerang gleich die Regierenden und werden zum Ausgangspunkt der Gretchenfrage eines jeden Wahljahres: Können sie es? Vertrauen wird nicht nur an Aussagen politischer Akteure gemessen, sondern an ihren Handlungen. Die Vielstimmigkeit und Multipolarität der föderalen Entscheidungsmechanismen führt zur regelmäßigen Frage, wer die Führung übernimmt, ob Angela Merkel die Kraft noch aufbringt und ob die Prozesse der Entscheidungsfindung zielführend sind. Politikjournalisten sezieren das offenkundige Machtvakuum: Merkel tritt ab, Laschet kommt nicht in Fahrt, und die Frage dominiert, ob er er Bundeskanzler kann?

## In Polittalkshows werden die Phasen der Berichterstattung sichtbar

Als eine der, wenn nicht als die offensichtlichsten Seismografen dieser Phasenorchestrierung können die Polittalkshows der öffentlich-rechtlichen Fernsehsender bezeichnet werden. In der Corona-Krise wurden sie zu einem Polit-Lagerfeuer für die gesamte Nation, in der Politikerinnen und Politiker wie in einer Pressekonferenz für Gesamtdeutschland ihre Leitlinien für die kommende Woche bekannt gaben und teils mit hehren Ideen bestückte Testballons zündeten, um zu prüfen, wie Verschärfungs- oder Lockerungsideen von einer breiten Bevölkerung aufgenommen werden. Die Polittalkshows machten bislang unbekannte Virologinnen zu Shootingstars der Pandemie. Auch deshalb, weil die immer gleichen Mediziner Woche für Woche eine Plattform bekamen, auf der sie sich gemeinsam mit den immer gleichen Regierungsvertretern – von der kaum eingeladenen Opposition unwidersprochen – über Zustandsbeschreibungen und Agenden die Bälle zuspielten. In der *Phase der Schockstarre *manövrierten Polittalkshows zwischen regierungsnaher Akteurszentrierung und Monothematisierung.

Die Inhaltsanalyse der Titel und Gästezusammensetzung von „Anne Will“, „hart aber fair“, „Maybrit Illner“ und „maischberger. die woche“ belegt diese Fokussierungstrends: In den 133 Sendungen des Jahres 2020 seit dem 27. Januar – an jenem Tag wurde der erste COVID-19-Fall in Deutschland berichtet – hatten 92 Ausgaben Corona zum Thema. Zunächst blieb das Virus weitgehend unbeachtet, die Talks drehten sich um die Regierungsbildung in Thüringen. Erst mit den steigenden Infektionszahlen ab März 2020 dominierte Corona und schaffte es daraufhin, alle anderen Themen vollständig zu verdrängen. Die Sendungstitel offenbaren zudem einen radikalen Wechsel von Sach- und Kritikorientierung im Zeitverlauf. Der Titel einer Sendung spiegelt die redaktionelle These, den roten Faden des Talks. Dieser rote Faden ist zu Beginn der Corona-Thematik sehr deutlich sachorientiert, was als Hinweis darauf verstanden werden kann, dem Informationsbedürfnis der Bevölkerung Rechnung zu tragen. Erst mit sinkenden Fallzahlen wird die Tonalität kritischer.

Sehr viel klarer wird das Bild mit Blick auf die Zusammensetzung der Gäste (n730) quer durch die Polittalkshows. Erwartungsgemäß kommen überwiegend Politikerinnen und Politiker zu Wort, unter ihnen dominiert die Bundesebene (58 %) vor der Landesebene (26 %) und weit abgeschlagen Kommunalvertreter (4 %). Außerdem in den Sesseln Platz nehmen durften andere Journalistinnen und Mediziner, davon der Großteil Virologen. Spannender noch ist die Verteilung nach Parteien: Die meisten Vertreterinnen entsenden durfte die Union (34 %), gefolgt von der SPD (29 %), den Grünen (12 %), der FDP (9 %) und der Linke (7 %), weit abgeschlagen die AfD mit 2,5 %. Da Polittalks oft die Debatte der vergangenen Woche abbilden und zuzuspitzen versuchen, kann dies als Hinweis auf die Rolle der jeweiligen Parteien im öffentlichen Diskurs insgesamt verstanden werden. Als auslösender Faktor denkbar ist gleichermaßen die Resonanzstärke der Aussagen bestimmter Parteien wie auch die Quantität und Qualität inhaltlicher Angebote für den öffentlichen Diskurs. Die Dominanz der regierungstragenden Parteien spiegelt sich interessanterweise immer dann an der Gästeauswahl der Polittalksendungen, wenn die Inzidenzen steigen. Bei „Anne Will“ beispielsweise blieb die Opposition im März de facto komplett und zum Jahresende hin weitgehend außen vor. Ausgeglichen dagegen das Einladungsszenario ab Mai im corona-gelockerten Sommer, hier bildet sich die *Phase der Rückbesinnung auf Kritik* sehr deutlich ab: Die Annalena Baerbocks und Christian Lindners der Republik finden wieder statt und dürfen die Regierenden vor sich hertreiben – bis die Zahlen wieder stiegen, und mit der zweiten Infektionswelle ein zweiter Schockstarren-Modus einsetzte. Die Gästeauswahl bildet mithin fast eins zu eins Inzidenzen und Notfall-Debatten ab. Je größer das Entscheidungsgewicht des „Merkel-Ministerpräsidenten-Kabinetts“, desto stärker der Fokus der Politikerklärer auf das Orakel Berlin-Mitte – und desto stärker parallel ein großes Polittalk-Schweigen der parlamentarischen Opposition.

Erst mit der Kritik um die Impfstoffbeschaffung der Europäischen Union und der Maskenaffäre in der CDU/CSU-Bundestagsfraktion folgt auch in den Polittalkshows eine neue Tonalität im Umgang mit den Corona-Politikern – es ist der Beginn der *Phase der parteipolitischen Akteurszentrierung: *Für die Regierungspolitik fallen nunmehr beginnender Wahlkampf im Superwahljahr und immer lauter werdende Kritik an der Corona-Politik zusammen. Die sonst Interviews vermeidende Bundeskanzlerin geht im März schließlich bei „Anne Will“ an die Öffentlichkeit und bekommt von der Moderatorin eine Stunde lang die Verfehlungen der Corona-Politik vorgehalten. Zu Beginn der Pandemie wäre eine solche Gemengelage undenkbar gewesen. Tage später muss sich NRW-Ministerpräsident Armin Laschet von Markus Lanz ins Kreuzverhör nehmen lassen, der ihn unerbittlich auf die vermeintlich schwelende Auseinandersetzung in der Union befragt: Corona-Politik und Bundestagswahlkampf verschwimmen zunehmend, innerparteilich wie in der medialen Debatte überlagern Wahlkampf und Parteipolitik nun Sachfragen um den Kampf gegen die Pandemie. Die Pandemie ist spätestens jetzt parteipolitisch geworden.

## Das große Durchstechen

Auf der Mikro-Ebene werden Prozesse politischer Kommunikation deutlicher denn je: Wir erleben das große Durchstechen. Jede vom Kanzleramt vorbereitete Vorlage ist längst publizistisch seziert, bevor die Runde der Ministerpräsidentinnen und -präsidenten sie überhaupt erfassen konnte. Und die mediale Erregung fordert vor allem „Mehr“ – mehr Verschärfung, Lockdown, Impfstoff, oder mehr Freiheit, Lockerung, Finanzhilfen (von Finanzminister Scholz 2020 als „Bazookas“ bezeichnet). Der Zugang zur Sphäre der politischen Entscheidungen indes ist partizipativer denn je: Pressekonferenzen werden online gestreamt, vorgeschaltet der stundenlange Blick auf leere Stühle im Bundeskanzleramt wie sonst nur auf den Schornstein der Sixtinischen Kapelle bei der Wahl eines neuen Papstes. Die Länge der Sitzungen wird zum Indikator für wahlweise Kontroverse oder Güte der Entscheidungen wie bei Tarifverhandlungen. Erst eine Nachtsitzung kann zum Durchbruch führen, diese Beobachtung wird zum dramaturgischen Effekt und bald schon zur Erwartungshaltung gegenüber den Teilnehmenden.

Wenn es aber nichts zu beschließen gibt in den vorbereiteten Papieren, bleiben ungeprüfte Schnellschüsse. Die vielzitierte „Osterruhe“ beispielsweise, eine nächtliche Idee aus der Sektion „gut gemeint, nicht gut gemacht“, und entsprechend schnell wieder abgeräumt. Merkel bittet um Verzeihung, erst die Öffentlichkeit, dann das Parlament. Wie notwendig transparente Kommunikation gegenüber der Öffentlichkeit ist, mag auch die Kanzlerin gemerkt haben, die sich zunächst regelmäßigen Interviews als zentralem journalistischem Mittel verweigerte und sich nur in akuten Krisensituationen kritischen Fragen stellte – sich stattdessen lieber von DB-Mobil als von journalistischen Leitmedien befragen ließ. Mit allen Mitteln kommunikative Kontrolle bewahren zu wollen, mag in normalen Zeiten gelingen; mittels Podcasts, vorgestanzten Web-Interviews des Bundespresseamts oder Pressekonferenzen im Verlautbarungsmodus. Die Pandemie jedoch erfordert kommunikative Präsenz. Wo Merkel eine Lücke lässt, wird sie von anderen gefüllt. Das Babylonische ist natürliche Folge des Schweigens im Kanzleramt, es führt zu Fragen an all jene, die sich ebenfalls berufen fühlen, bisweilen auch zu mehr als ihrer aktuellen Aufgabe. Merkels letzter publizistischer Joker: „Anne Will“. Die Flucht nach vorne im Einzelinterview, Einschaltquoten garantiert, Sofa-Modus und gemäßigter Ton. Weitere Gäste etwa aus der Opposition musste sie nicht fürchten, durfte stattdessen mehrfach eine unverhohlene Drohung in Richtung Bundesländer wiederholen: Sollten die Länder nicht liefern, regelt es der Bund, Infektionsschutzgesetz sei Dank. Ein bisschen Ausnahmezustand im Talkformat, dann ist auch dieser Auftritt Geschichte.

Was bedeutet es für die Demokratie, wenn Vertraulichkeit als Faktor der Entscheidungsfindung aufgehoben ist? Eine nie dagewesene Echtzeit-Kommunikation mit mehr Möglichkeiten zur aktiven Partizipation. Politikerinnen machen ihre eigene Verunsicherung deutlich, ihre Beratungsabhängigkeit nicht nur von Virologen. Zugleich zeigt sich eine krisenbedingte Akteurszentrierung der journalistischen Berichterstattung.

Die Pandemie wird dominiert von einem kommunikativen Kammerflimmern, inklusive Verunsicherung und negativer Gefühle. Bisweilen aufgrund allerlei verkürzter und falsch verstandener Push-Meldungen wie „Merkel will kompletten Stopp von öffentlichem Nahverkehr“, was sie so nie gesagt hat. Dann aber befeuert vom Zickzack-Kurs politischer Akteure, Beispiel: Eil-Meldung „Schulen in Dortmund schließen“, gefolgt von Eil-Meldung „Landesregierung untersagt Schulschließung in Dortmund“. Wo gehobelt wird, fallen Späne, könnte nun manch einer einwerfen. Doch ist damit die Gefahr verbunden, dass die Ticker-Dominanz am Vertrauen in Qualitätsjournalismus und politischen Entscheidungsprozessen gleichermaßen rüttelt. Die Rolle von Politikjournalistinnen und -journalisten bei der Filterung oder Themen-Selektion für politische Entscheidungsrunden im Echtzeit-Modus ist relevanter denn je. In der Kurztaktigkeit der Corona-Entscheidungen blieb scheinbar keine Zeit für regelgeleitete Meinungsbildungsprozesse. Politikjournalismus vermag in diesem Kontext gleichermaßen Sensor oder Impulsgeber gesellschaftlicher Stimmungen sein. Die Herausforderung für Politikjournalismus in der Langzeit-Pandemie wird sein, Kritik und Kontrolle sachorientiert zu fokussieren und trotz des Vorwahlkampfs nicht allein Akteurinnen und Akteure in den Mittelpunkt zu rücken. Aktuell erleben wir noch das genaue Gegenteil: War es im letzten Jahr das Entsetzen über einen Journalismus, der in Krisenzeiten plötzlich seinen Kernauftrag – Kritik und Kontrolle – zu vergessen schien, dominiert nunmehr ein Qualitätsjournalismus, der politische Entscheidungsträgerinnen vor sich hertreibt und damit aus Akteuren Re-Akteure macht. Das allerdings liegt wohl mehr an der Politik als an den Medien. Aus einer gesundheitlichen Krise ist nach Planlosigkeiten und Korruptionsdiskussionen eine politische Krise geworden, in der die kollektive Richtungssuche um den richtigen Umgang mit Corona durch einen klassischen Grabenkampf zwischen politischen Personen und Parteien ersetzt wurde, und der von den Medien nun so kritisch-süffisant begleitet wird wie in Nicht-Krisenzeiten; natürlich mit den bekannten Mechanismen der Fokussierung auf Personaldebatten und parteiinterne Scharmützel – aber so, dass sämtliches Regierungshandeln nun wieder diskursiv auf dem Prüfstand der Öffentlichkeit steht. Während Qualitätsjournalismus also zurückgefunden hat zu den ureigenen Aufgaben, sind wesentliche politische Akteure noch mit sich selbst und ihrer Zukunft beschäftigt.
